# Patient experiences of muscle biopsy in idiopathic inflammatory myopathies: a cross-sectional survey

**DOI:** 10.1007/s00296-024-05668-4

**Published:** 2024-07-31

**Authors:** Benjamin Sutu, Samuel Maxwell, Shereen Oon, Laura Ross, Jessica Day

**Affiliations:** 1 Department of Rheumatology, The Royal Melbourne Hospital, Melbourne, Australia; 2 Inflammation Division, The Walter and Eliza Hall Institute of Medical Research, Melbourne, Australia; 3 Department of Rheumatology, St Vincent’s Hospital Melbourne, Melbourne, Australia; 4 Department of Medicine, The University of Melbourne, Melbourne, Australia; 5 Department of Medical Biology, The University of Melbourne, Melbourne, Australia

**Keywords:** Myositis, Biopsy, Diagnosis, Patient-centered care, Patient preference, Surveys and questionnaires

## Abstract

Introduction: Muscle biopsy plays an important role in the diagnostic evaluation of individuals with suspected idiopathic inflammatory myopathies (IIM). However, variability in biopsy practices may result in a heterogenous patient experience. The existing literature offers limited insights into the experiences and perspectives of patients undergoing muscle biopsy. Methods: This study employed a 27-item online survey to comprehensively characterise the experience of muscle biopsy among Australian patients, including their concerns, beliefs about procedure utility, information sources, physical sensations, perceived complications and recovery. Results: A total of 111 Australian individuals who reported a diagnosis of IIM completed the survey, with data collected from March to June 2023. Most participants had inclusion body myositis (76/111, 68.5%) and had undergone one biopsy procedure (87/111, 78.4%) as part of their IIM work-up. Nine of the 111 respondents did not undergo a muscle biopsy. The procedure was well-tolerated by many respondents, however, a notable number of respondents experienced post-procedural pain lasting > 72 h (27/102, 26.5%), increasing weakness post-biopsy (13.7%), numbness at the biopsy site (18/102, 17.6%) and a recovery time beyond 3 days (36/102, 35.3%). A substantial minority (30/111, 27%) felt they were inadequately informed about the risks and benefits of the procedure. Conclusions: This survey highlights that although muscle biopsy is often well-tolerated, there are considerable patient concerns that are often inadequately addressed. Our findings underscore the need for improved patient-doctor communication and support throughout the biopsy process.

**Supplementary Information:**

The online version contains supplementary material available at 10.1007/s00296-024-05668-4.

## Introduction

Idiopathic inflammatory myopathies (IIM) are a group of multi-system diseases characterised by a predilection for skeletal muscle inflammation. This heterogenous group includes dermatomyositis (DM), polymyositis (PM), anti-synthetase syndrome (ASyS), overlap myositis (OM), immune-mediated necrotising myopathy (IMNM) and inclusion body myositis (IBM). Although rare, with a prevalence estimated between 5.8 and 9.7 per 100,000 people [1] depending on IIM subtype, these diseases can lead to significant morbidity, mortality and utilisation of healthcare resources [2]. Timely diagnosis and treatment are important to prevent adverse outcomes and ensure best patient-centred care experiences.

The diagnosis of IIM can be made based on a combination of clinical manifestations, which may include muscle weakness, serological findings, electromyography, imaging, and muscle biopsy features. While autoantibody profiling has emerged as an important diagnostic tool, nearly 40% of IIM patients are seronegative [3] and concerns persist about the performance of the myositis immunoblot [4–9]. Moreover, while IIM subtypes may share common clinical features, they can have unique histopathological features. Thus, muscle biopsy remains a vital test to assist in IIM diagnosis and subtyping.

A variety of muscle sampling practices have been reported worldwide, suggesting that the optimal method of obtaining muscle tissue remains unknown [10–16]. Reported techniques include open surgical biopsy [10–12], percutaneous or semi-open needle biopsies, and conchotome forceps procedures [13, 14]. While these approaches involve different surgical instruments and yield varying amounts of muscle, available data suggest muscle biopsy to be a safe, well-tolerated procedure, regardless of technique [13, 17, 18].

While observational cohort studies provide insights into the clinical outcomes and medical complications of muscle biopsy, there is a noticeable lack of data regarding the perspective of the patients who undergo these procedures. It is increasingly recognised that research studies and initiatives to improve healthcare services must include an assessment of what matters most to patients. Muscle biopsy, like any clinical investigation, should be tolerable and present an acceptable risk-benefit profile for the individual undergoing the procedure. Exploring the patient perspective helps shed light on concerns, experiences, logistical difficulties, and adverse patient impacts. In this study, we explore the experience and perceptions of muscle biopsy for Australian patients with IIM by gathering insights directly from patients themselves. Such knowledge helps tailor information resources for people with suspected IIM undergoing these procedures, in addition to patient-centred guidelines regarding performance of muscle biopsy procedures.

## Methods

### Participants

Individuals from Australia with a self-reported diagnosis of “myositis” were eligible to participate in this study. This included all forms of adult and juvenile immune-mediated muscle inflammatory disorders, including dermatomyositis (DM), polymyositis (PM), anti-synthetase syndrome (ASyS), overlap myositis (OM), immune-mediated necrotising myopathy (IMNM) and inclusion body myositis (IBM). Participants were invited to complete a voluntary electronic survey via the REDCap (Research Electronic Data Capture) platform hosted at the Royal Melbourne Hospital (RMH) Health Intelligence Unit. Informed consent was obtained via a cover page embedded within the survey and no incentives were offered for survey completion. Although participants were given the option to answer the survey anonymously, they could choose to provide their name and email address if they wished to receive updates on the study or contribute to future surveys.

### Survey design

A 27-item multi-page web-based survey (Supplementary Fig. 1) was developed to capture basic demographics, healthcare practitioner involvement in diagnosis, muscle biopsy details, and patient perceptions on receiving and obtaining information about muscle biopsy from healthcare professionals. Most questions (17) were multiple choice with a single answer option, while some (6) allowed participants to select multiple answers. An ‘other (please specify)’ option was provided for one item. At the end of the survey, participants were given the opportunity to provide comments or feedback via a free-text window. The survey was presented on a web-based platform that required participants to scroll across multiple pages. An “incomplete” response was considered as one which did not have any information entered beyond the first page of the questionnaire. These responses were removed from further analysis.

The survey was developed in consultation with the Myositis Discovery Programme Consumer Panel. The questions were informed by group discussions with 18 consumers and a previously published systematic review of muscle biopsy practices [13]. Pretesting was conducted with the Myositis Consumer Panel, where a draft survey was presented and questions were refined through open discussions. The survey did not exclude participants with myositis who had not undergone a muscle biopsy, as their reasons and perceptions about not proceeding with biopsy were felt to be informative. To ensure accuracy, the survey underwent multiple rounds of dummy fill-ups by consumers and clinicians to identify errors in wording, grammar, and syntax. Pilot testing was conducted by 4 consumers and 3 clinicians to identify issues with the survey. Subsequent rounds of dummy fill-ups were performed by the clinicians prior to release of the survey. The revised survey was sent to the Myositis Discovery Programme Consumer Panel and the International Myositis Assessment & Clinical Studies Group Scientific Committee for feedback and approval. Repeat pilot testing was performed after incorporating all feedback to ensure the integrity of the survey.

Ethics approval was obtained on the 14th September 2022 from the Melbourne Health Human Research Ethics Committee (Melbourne, Australia – Protocol # 2022.153).

### Sampling strategy

The survey was circulated via email lists of the Myositis Association of Australia, a patient consumer group comprising 440 members. Contact details for the administrators of this group were identified through previous interactions with the authors. Snowball sampling was employed, whereby participants were invited to forward the survey link to other individuals who had been diagnosed with IIM. The survey link was open from 1st March 2023 to 31st June 2023.

### Analysis

Survey responses were analysed using descriptive statistics. Categorical variables were presented as frequencies and proportions. Free text feedback was analysed through a streamlined thematic analysis process, consisting of iterative readings, coding and thematic organisation. Key quotations exemplifying these themes were selected for inclusion in the manuscript.

This study adhered to the CHERRIES checklist as the basis for survey reporting guidance [19, 20].

## Results

One-hundred and fifty-four individual responses were collected during the sampling period. Of these, 43 responses were incomplete and were excluded from further analysis. Seventy-four patients provided commentary in the free text section. Baseline characteristics of included participants are presented in Table 1. The majority of respondents had a diagnosis of IBM (68.5%), followed by polymyositis (10.8%) and dermatomyositis (8.1%). Few respondents had necrotising myositis, overlap myositis or ASyS. Most respondents had long-standing disease (83% had > 3 years disease duration). Most respondents were under the care of a neurologist (76.6%) or rheumatologist (32.4%) for management of their IIM.

**Table 1:** Participant characteristics

Diagnosis	Response, (%, *N*)
Dermatomyositis	8.1% (9/111)
Juvenile dermatomyositis	0% (0/111)
Polymyositis	10.8% (12/111)
Inclusion body myositis	68.5% (76/111)
Anti-synthetase syndrome	2.7% (3/111)
Overlap myositis	0% (0/111)
Necrotising myositis	3.6% (4/111)
Other	3.6% (4/111)
Unsure	2.7% (3/111)
**Medical Professional responsible for care of IIM**	**Response (%**,** N)**
Rheumatologist	32.4% (36/111)
Neurologist	76.6% (85/111)
Internal Medicine/General Medicine	2.7% (3/111)
Respiratory Physician /Pulmonologist	2.7% (3/111)
Primary Care/Family Doctor/General Practitioner	23.4% (26/111)
Rehabilitation Physician	0% (0/111)
Unclear	1.8% (2/111)
Other specialist ◊	12.6% (14/111)
**Disease duration at time of survey**	**Response (%**,** N)**
> 22 years	6.3% (7/111)
Between 12–22 years	25.2% (28/111)
Between 3–12 years	51.4% (57/111)
< 3 years	17.1% (19/111)
**Number of muscle biopsies per respondent**	Response *%,** N)**
0 biopsies	8.1% (9/111)
1 biopsy	78.4% (87/111)
2 biopsies	13.5% (15/111)
≥3 biopsies	0% (0/111)

* - participants were permitted to enter in more than one response

◊ - further details available in Supplemental Data section

### Indications for muscle biopsy

Most participants (92%, 102/111) underwent a muscle biopsy as part of their diagnostic assessment. Of the nine participants who did not undergo a muscle biopsy, five reported that a muscle biopsy was not offered or was deemed unnecessary by their treating doctor. One participant reported that their myositis was diagnosed through a cutaneous lesional biopsy, and another reported that a muscle biopsy was suggested but the medical risks were deemed too high to proceed. One participant could not recall the reason why they did not have a muscle biopsy while another reported that muscle biopsies were not available in their local area, and they were unable to travel to another location to pursue a biopsy.

For the fifteen participants who had more than one muscle biopsy, a notable number (5/15, 33.3%) reported that a further biopsy was required as their index biopsy provided insufficient clinical information. Four (26.7%) reported that a second muscle biopsy was performed for disease monitoring while another four (26.7%) had multiple biopsies for research purposes. Two patients were uncertain why they had multiple biopsies performed.

### Procedural aspects of muscle biopsy

The most common site for muscle biopsy was the quadriceps (85.3%) followed by the biceps/triceps (16.7%) and deltoid (4.9%) (Table 2). Rarer sites included the gastrocnemius, tibialis anterior, hamstrings, brachioradialis muscle, paraspinal muscles and the adductor femoris. Several participants expressed concern about muscle selection process, highlighting communication gaps between the treating clinician and the proceduralist, leading to an incomplete understanding of their medical history by those performing the procedure:

**Table 2:** Muscle biopsy characteristics

Muscle group sampled	Response (%, *N*)
Quadriceps	85.3% (87/102)
Hamstrings	1.0% (1/102)
Tibialis anterior	1.0% (1/102)
Gastrocnemius	1.0% (1/102)
Biceps/Triceps	16.7% (17/102)
Deltoid	4.9% (5/102)
Unclear/Unsure	1.0% (1/102)
Other	2.9% (3/102)
**Anaesthetic status**	**Response (%**,** N)**
“Awake”; Loco-regional anaesthesia/sedation	80.4% (82/102)
“Asleep”; General anaesthesia	15.7% (16/102)
Unclear/unsure	2.9% (3/102)
No response	1.0% (1/102)
**Proceduralist**	**Response (%**,** N)**
Surgeon	73.% (75/102)
Radiologist	0% (0/102)
Rheumatologist	4.9% (5/102)
Neurologist	8.8% (9/102)
Unclear/unsure	12.7% (13/102)
**Biopsy type**	**Response (%**,** N)**
Open biopsy	73.5% (75/102)
Needle biopsy	2.0% (2/102)
Conchotome forceps biopsy	2.9% (3/102)
Unclear/unsure	10.8% (11/102)
**Biopsy setting**	**Response (%**,** N)**
Day procedure	75.5% (77/102)
Inpatient	24.5% (25/102)
*For participants who were admitted (hospital inpatient) for their procedure*,* they were asked to respond with their length of stay*	
**Length of stay**	**Response (%**,** N)**
< 72 h	56% (14/25)
> 72 h	44% (11/25)

λ - participants were permitted to enter in no responses or multiple responses

*“The Neurosurgeon…only took samples from muscles normally biopsied*,* not the ones identified under my MRI. This led to lengthy*,* delayed testing for over 3 months to finally reach a diagnosis.”* – Participant 32.

*“I believe there could have been better coordination between the health professionals involved in my biopsy. I experienced some anxiety at what I believed to be an incomplete transfer of information about the site of the biopsy. I took action to ensure the site of the biopsy was correctly understood by the surgeon.”* - Participant 67.

The majority of participants reported having an open biopsy (68.6%) performed by a surgeon (73.5%) (Table 2). A quarter of respondents underwent muscle biopsy as part of an inpatient admission, while for the remainder this test was performed as a day procedure. Most respondents recalled being “asleep” (80.4%) during their biopsy procedure – suggesting they were performed using anaesthesia or sedation. Of the 82 participants who recalled being “asleep” for their procedure, 12 (14.6%) reported that they were intubated, 36 (43.9%) participants reported not being intubated, whereas 34 (41.5%) could not recall. For 16 respondents, the muscle biopsy was performed with the participant being “awake”.

### Experiences during and post procedure

The biopsy procedure was well-tolerated by many respondents, with 56% reporting no negative sensations during the event and 45.1% reporting no adverse events following the procedure (Table 3). However, a number of respondents experienced pain associated with their procedure (13.7%), post-procedural pain for > 72 h (26.5%) or post-procedural pain that required analgesia (35.3%). Increased weakness (13.7%) and numbness at the biopsy site (17.6%) were commonly reported. Two-thirds (67%) of all respondents who underwent a muscle biopsy reported a scar > 2 cm from their procedure.

**Table 3:** Participant experiences with muscle biopsy

Symptom experienced during muscle biopsy	Response (%, *N*)
Pain	13.7% (14/102)
Uncomfortable pressure	4.9% (5/102)
Lightheadedness	0% (0/102)
Anxiety	8.8% (9/102)
Other sensation	0% (0/102)
No negative sensations	55.9% (57/102)
Unsure/cannot recall	8.8% (9/102)
**Adverse event experienced after muscle biopsy**	**Response (%**,** N)**
Bleeding	2.9% (3/102)
Bruising	3.9% (4/102)
Pain requiring analgesia	35.3% (36/102)
Pain lasting more than 72 h	26.5% (27/102)
Numbness	17.6% (18/102)
Increased weakness of biopsied muscle lasting > 21 days	13.7% (14/102)
Scarring	8.8% (9/102)
No adverse events	45.1% (46/102)
Other event	4.9% (5/102)
**Length of scar**	**Response (%**,** N)**
No scar	21.6% (22/102)
Less than 1 cm	4.9% (5/102)
1–2 cm	6.9% (7/102)
2–3 cm	31.4% (32/102)
Greater than 3 cm	35.3% (36/102)
**Time taken to resume normal duties post procedure**	**Response (%**,** N)**
1 day	31.4% (32/102)
2 days	19.6% (20/102)
3 days	13.7% (14/102)
More than 3 days	35.3% (36/102)
**Time taken off work post procedure**	**Response (%**,** N)**
Yes	22.5% (23/102)
No	24.5% (25/102)
Not employed at time of biopsy	52.9% (54/102)

* - participants were permitted to enter in more than one response

λ - participants were permitted to enter in no responses or multiple responses, influencing the overall percentage of positive responses

A significant proportion of patients experienced a moderate recovery period, with 35.3% of participants taking more than 3 days to return to their normal level of function, and 47.9% (23/48) of those in paid employment at the time of their biopsy needing to take time off work. For those who required time off work, the median duration was 4 days (range 1-120 days, interquartile range of 2–7 days). Several participants provided commentary on their experience of prolonged recovery:

*“I do not want more tissue removed as it took months to fully recover functionally from the first biopsy”* – Participant 5

*“I was not made aware of the duration of recovery but was longer than I anticipated. I have had ongoing discomfort at the incision site. I would not be prepared to have another muscle biopsy if it involved the same procedure.”* – Participant 70.

*“Despite the small incision and tissue sample*,* I experienced quite a lot of discomfort for some days”*. – Participant 54.

Conversely, others reported a favourable response to the procedure:

*“The procedure was better than expected*,* I only experienced slight discomfort”* – Participant 51.

### Receiving and obtaining information about muscle biopsy

While the majority of participants (73%) believed they were provided sufficient information before their biopsy, a substantial minority (27%) of participants felt that they were not provided with adequate information about the process or given sufficient opportunity to ask questions about the risks and benefits of the procedure (Table 4).

**Table 4:** Participants’ perceptions on the muscle biopsy experience

Was adequate information provided about the risks and benefits of the procedure?	Response (%, *N*)
Yes	73% (81/111)
No	27% (30/111)
**Patient concerns about undergoing muscle pre-biopsy**	**Response (%**,** N)**
Pain	27.0% (30/111)
Wound infection	19.8% (22/111)
Scar	11.7% (13/111)
Muscle or nerve damage	23.4% (26/111)
Increased weakness	18.9% (21/111)
Complications of general anaesthesia	15.3% (17/111)
Time off paid employment	8.1% (9/111)
Financial cost of muscle biopsy	1.8% (2/111)
Transport to and from procedure	10.8% (12/111)
Stay in hospital/inpatient admission	6.3% (7/111)
No concerns	40.5% (45/111)
Other concerns	9.0% (10/111)
**Source of information for patients regarding muscle biopsy risks and benefits.**	**Response (%**,** N)**
Diagnosing doctor	57.7% (64/111)
Proceduralist	39.6% (44/111)
Family doctor/Primary care doctor/General practitioner	8.1% (9/111)
Patient support group	4.5% (5/111)
Social media	2.7% (3/111)
Family and friends	0.9% (1/111)
No information sought	23.4% (26/111)
Other	3.6% (4/111)

* - participants were permitted to enter in more than one response

While 40.5% of participants recalled having no concerns about the muscle biopsy prior to undergoing the procedure, among those who did, the common concerns included pain (27%), muscle or nerve damage (23.4%), wound infection (19.8%) or increased weakness (18.9%). Most participants sought information about muscle biopsies from the doctor responsible for diagnosing their condition (57.7%), while others asked the proceduralist (39.6%). 23% of respondents did not seek out any further information while only a small number sought information from fellow patients (4.5%) or social media (2.7%). Several patients commented on a lack of communication from their treating specialists or encountered conflicting opinions from various information sources:

*“I thought I would be having a needle biopsy*,* although to be fair my neurologist did not describe what procedure I would have. It was only in the pre-op area that the surgeon said that he would be taking a 1 cm cube of tissue as pathologists preferred a larger sample and needle biopsies did not always give a suitable sample.”* – Participant 6

*“A Myositis Association Australia member communicated her debilitating biopsy experience but I formed the view it could not be that bad - it was only a biopsy. She was right.”* – Participant 39.

### Beliefs about the utility of muscle biopsy

Participants were asked to comment on whether they felt a muscle biopsy was “helpful” to the overall diagnosis and management of their myositis. While the majority (90/102, 88.2%) believed their biopsy was helpful to their overall management, there were six (5.9%) who did not believe the biopsy was helpful, and a further six (5.9%) who were unclear about the overall utility of undergoing a biopsy (Table 3).

### Patient expectations compared to their experience

Of the 102 participants who underwent a muscle biopsy procedure, many reported their muscle biopsy experience was either better than expected (19.6%, 20/102) or consistent with expectations (53.9%, 56/102). However, approximately one quarter of respondents (25.5%, 26/102) reported their muscle biopsy experience was worse than expected. All participants who reported a worse muscle biopsy experience than expected also reported experiencing complications. The most commonly reported complication was post-procedural pain lasting greater than 3 days, as reported by 61.5% of the 26 patients who believed their biopsy experience was worse than expected.

### Engagement with research

Only 19.8% (22/111) of participants indicated they would consider donating a muscle biopsy sample if requested for research purposes. Most participants willing to donate muscle samples for medical research commented that their motivation was to provide more information to improve treatment and overall disease management outcomes.

*“I … would very much like to assist with better diagnosis*,* understanding and awareness of IBM”* – Participant 9.

## Discussion

Although muscle biopsy has long been considered an important tool in the diagnosis of IIM, there is a notable lack of data regarding the patient experience of this invasive procedure. While several studies have evaluated post-procedural complication rates [21–23] and peri-procedural pain scores [24–26], this study marks a significant step forward as the first systematic evaluation of patients’ perceptions, beliefs and experiences related to muscle biopsy in the evaluation of IIM.

Overall, our study suggests that muscle biopsy is well-tolerated by many individuals as a diagnostic test for IIM. However, our findings also highlight a disconnect between some patients’ expectations of the muscle biopsy procedure, and their actual experience. Approximately one quarter of participants indicated that their muscle biopsy experience was worse than anticipated, suggesting that they may not have been adequately informed or counselled about the procedure. Poor communication may be a key contributing factor with 27% of participants reporting they did not receive sufficient information from their healthcare providers prior to their muscle biopsy. Several participants also raised concerns about the lack of coordinated communication between their diagnostician and their biopsy proceduralist, which led to anxiety and distress that the wrong muscle was or would be biopsied. Inadequate communication may also explain why nearly 12% of participants were uncertain about the biopsy findings.

Many participants had concerns about the potential risks of the muscle biopsy, and it is unclear if these concerns were adequately addressed prior to the procedure. It is notable that few participants sought information about muscle biopsy from sources other than their diagnostician or proceduralist. This emphasises the crucial importance of healthcare professionals in providing patients adequate time and information to address their concerns.

Our study revealed diversity in the patient experience of muscle biopsy, indicating that approaches to these procedures are not standardised across Australia. This heterogeneity encompassed all aspects of the muscle biopsy process, from the perceived quality of information provided to the patient regarding biopsy risks and benefits, to procedural methods used, the type of anaesthetic administered, and whether the procedure was performed as an inpatient or outpatient. It is noteworthy that the majority of our survey participants had IBM and yet heterogeneity of experience was still observed.

Various muscle biopsy approaches have been reported in the literature [13–16] and local variation can be expected based on proceduralist and institutional preference. While previous studies have concentrated on the diagnostic performance of diverse muscle biopsy methodologies [26, 27], our research underscores the significance of patient acceptability, confidence and comfort. It is worth noting that the majority of participants in our study underwent open surgical biopsies, which involve larger incisions and result in more substantial tissue samples compared to percutaneous biopsies. Thus, we cannot draw firm conclusions about the experience of percutaneous biopsy relative to the open surgical approach. To achieve the best possible patient care outcomes, future efforts to evaluate and compare muscle biopsy approaches should not focus solely on diagnostic efficacy but must also consider the patient perspective. By incorporating such perspectives, procedures can be better aligned to patient expectations and deliver a more patient-centred healthcare experience. Our survey results highlight the importance patients place on symptoms like pain and physical deconditioning following their procedure. Improved models of care may include improved pre-procedure counselling and post-acute care planning, including proactive referrals to Allied Health to improve functional recovery post-procedure, and more comprehensive pain education and management. More comprehensive pre-procedural planning may alleviate some of the anxiety patients have about their procedure and help them move through the post-procedural process feeling better supported and more well-equipped.

Though the current literature suggests that muscle biopsy complication rates are low [13, 28–32], our study highlights that some patients undergoing these procedures experience more morbidity than commonly acknowledged. The rates of pain and perceived complications were notably higher than the rates reported in the medical literature [12, 13, 21–23, 25]. Our study underpins the need to better identify patients at higher risk of post-procedural complications and poor recovery. Some patients in our study reported increased weakness following a muscle biopsy, and a substantial number experienced protracted recovery. These findings may reflect post-procedural deconditioning [33], a pertinent concern given that individuals with IIM often have compromised muscle reserve. Identifying those at risk of such outcomes is important, as better preparations may be made for their post-procedural care and subsequent rehabilitation. Future research could explore the effects of proactive interventions and improved communication when managing high-risk patients in order to better mitigate potential negative psychosocial and physical impacts.

### Limitations

There are several limitations to this study. As a questionnaire-based study distributed online that utilised snowball sampling, it carries the potential for selection bias, capturing the experiences of a select cohort of participants with IIM willing to offer their insights and perspective. It is possible that those with negative experiences or stronger opinions were more inclined to participate in the survey and provide free-text comments [34].

The small sample size and relative lack of diversity, with most respondents having IBM, diminishes our ability to draw firm conclusions for the broader myositis patient population. Notably, no participants with juvenile forms of idiopathic inflammatory myopathies responded to our survey. Future studies should aim to capture this cohort as there are unique issues relating to muscle biopsy procedure in this group [35, 36].

Another limitation is the reliance on participant’s self-reported diagnosis. This survey was disseminated through a consumer group to increase reach, meaning it was not possible to verify self-reported diagnoses. This is an inherent limitation of patient-reported data.

The survey did not capture specific data regarding the indication of initial biopsy, assuming it was conducted for diagnostic evaluation since biopsy is the gold standard diagnostic test for myositis. However, reasons for not undergoing a biopsy and indications for undergoing a second biopsy were recorded. Delays to biopsy were not captured in this survey, which is another limitation to this study.

The questionnaire was only available to Australian participants, which limits the study’s generalisability, considering the global diversity in muscle biopsy practices [13, 15]. Further, the survey was only available in English, therefore we were unable to reflect on the experience of non-English speaking individuals. The electronic format also preferentially engaged technologically-proficient respondents, thus introducing potential selection bias. Finally, the questionnaire relied on participant recall. Future studies would be enriched by capturing contemporaneous patient experiences and medical record data.

## Conclusion

Patients with IIM typically undergo a muscle biopsy as part of their diagnostic evaluation, yet the patient experience of these procedures is poorly documented. Our study represents the most comprehensive evaluation of the patient experience of muscle biopsy within the context of IIM. The majority of patients had an acceptable experience, however the experience is worse than anticipated for a notable minority. We have identified suboptimal communication as a potential contributor to a mismatch between patient expectations and the actual muscle biopsy experience. Enhancing patient-doctor communication and identification of patients at risk for complications are important steps to improve the overall muscle biopsy experience for patients.

## Electronic supplementary material

Below is the link to the electronic supplementary material.

Supplementary Material 1

Supplementary Material 2

Supplementary Material 3

Supplementary Material 4

## Data Availability

Data are available from the corresponding author upon reasonable request.

## Declarations

### Conference abstract publication

Abstract publication at 2024 Joint ARA and NZRA Annual Scientific Meeting: Sutu B, Maxwell S, Oon S, Ross L and Day J, “P051: Patient experiences of muscle biopsy: A survey of Australian patients with idiopathic inflammatory myopathy” *International Journal of Rheumatic Diseases*, Volume 27 Special Issue: 2024 Joint ARA and NZRA Annual Scientific Meeting, 18–21 May 2024, Te Pae, Christchurch, New Zealand. (doi: 10.1111/1756-185X.15172).

## Abbreviations

ADL: Activities of daily living
ASyS: Anti-synthetase syndrome
DM: Dermatomyositis
IBM: Inclusion body myositis
IIM: Idiopathic inflammatory myositis
IMNM: Immune-mediated necrotising myopathy
MRI: Magnetic resonance imaging
OM: Overlap myositis
PM: Polymyositis
REDCap: Research Electronic Data Capture
RMH: Royal Melbourne Hospital
SD: Standard deviation
