# Supplementary material for: Patient experiences of muscle biopsy in idiopathic inflammatory myopathies: a cross-sectional survey
Source: Rheumatol Int. 2024 Jul 31;44(10):2129–37. doi: 10.1007/s00296-024-05668-4 (PMC11393206; doi:10.1007/s00296-024-05668-4)
Supplement: Supplementary file 2 — Supplementary Material 2 [file 296_2024_5668_MOESM2_ESM.docx]

**Supplemental Data**

| Participants were asked to identify the medical professional/s involved in the care of their myositis. Some participants identified “other” medical specialists who were involved in their care at the time of diagnosis. Participants who entered this option were invited to identify the specialty of the healthcare professional. | | |
| --- | --- | --- |
| **Medical Professional** | **Response** | **Percentage** |
| Dermatologist | 1/14 | 7.1% |
| Geriatrician/Gerontologist | 1/14 | 7.1% |
| Neurosurgeon | 2/14 | 14.3% |
| Immunologist | 4/14 | 28.6% |
| Cardiologist | 1/14 | 7.1% |
| Gastroenterologist | 1/14 | 7.1% |
| Non-medical specialist (physiotherapist) | 1/14 | 7.1% |
| Specialist, not otherwise specified | 3/14 | 21.4% |

Supplemental table 1.1: Further information about specialists involved in participants’ care

| Participants were asked to identify the date they were first diagnosed with myositis (participants were asked to enter in their best recollection in date, month and year) | | |
| --- | --- | --- |
| **Time Period** | **Response** | **Percentage** |
| Before 2000 | 3/111 | 2.7% |
| Between 2000-2010 | 13/111 | 11.7% |
| Between 2011-2020 | 64/111 | 57.7% |
| After 2020 | 31/111 | 27.9% |

Supplemental table 1.2: Date of first diagnosis of myositis separated into time periods
