# Supplementary material for: Patient experiences of muscle biopsy in idiopathic inflammatory myopathies: a cross-sectional survey
Source: Rheumatol Int. 2024 Jul 31;44(10):2129–37. doi: 10.1007/s00296-024-05668-4 (PMC11393206; doi:10.1007/s00296-024-05668-4)
Supplement: Supplementary file 3 — Supplementary Material 3 [file 296_2024_5668_MOESM3_ESM.docx]

**Supplemental Figure 1**

**Survey questionnaire via REDCap**

**Patient Experiences of Muscle Biopsy – a survey study**

Thank you for showing interest in this research study. Below is relevant information pertaining to the study. Please read all information before proceeding. If you have any questions about this study please contact Dr Jessica Day.

**Who is conducting the study?**

The principal investigator of this study is Dr Jessica Day, a rheumatologist and clinician scientist at the Royal Melbourne Hospital and Walter and Eliza Hall Institute of Medical Research (Melbourne, Australia).

**Why is the study being conducted?**

Muscle biopsy is a commonly performed procedure in patients with suspected myositis, however muscle biopsy practices and techniques vary widely between institutions. The purpose of this survey is to explore the experiences of patients with myositis with respect to muscle biopsy. You are encouraged to participate even if you haven't had a muscle biopsy. The information obtained here may ultimately help develop recommendations regarding muscle biopsy practices in evaluation of this disease.

Please note: the definition of 'myositis' for the purposes of this survey includes all forms of adult and juvenile dermatomyositis (DM), polymyositis (PM), anti-synthetase syndrome (ASyS), overlap myositis (OM), immune-mediated necrotising myopathy (IMNM) and inclusion body myositis (IBM) - in summary any immune-mediated muscle inflammation.

**What is expected of you as a research participant?**

Participants are expected to provide an answer to each question on the survey, which takes about 15 minutes to complete. It is optional to state your name and email address. If you do, we may contact you regarding future surveys and developments regarding muscle biopsy and diagnostic evaluation in patients with myositis.

**Risks and benefits:**

There are no risks associated with this study. The direct individual benefits for participation are minimal. Nonetheless, an enhanced understanding of muscle biopsy practices worldwide may facilitate future guidelines or recommendations regarding this procedure. You will not be paid nor will there be any costs to you for participating in this survey.

**Voluntary participation/withdrawal:**

Your participation in this study is voluntary and you may stop answering the questions at any time. However once you complete the survey you cannot remove your information as the researchers will not know which information is yours. You may be withdrawn from the study if you do not follow the instructions for participation.

**Confidentiality:**

Please note it is optional to state your name and email address. If you choose to provide us with identifiable information this will be stored in a password protected account in a REDCap database, accessible only to Dr Jessica Day and research staff assigned to this project. Identifiable information will be stored separately from your survey response information so that your specific survey responses are not identifiable. If you provide your contact details, you are giving consent for study investigators to store your contact details and you may be contacted again with further surveys regarding muscle biopsy practices and diagnostic evaluation of myositis. The results of this study may be published and shared with other members of the medical and scientific community. Your personal information and responses to the survey will remain confidential. Data will be stored for 5 years. **Please note that the country of residence of respondents may be published**. Given myositis is a rare condition, please consider whether your country of residence may identify you before providing us with this information.

**Ethics Approval:**

Ethics approval for this study was obtained by the Melbourne Health Human Research Ethics Committee, Melbourne, Australia (Ethics Protocol # 2022.153).

**Consumer Involvement:**

The study investigators would like to acknowledge the valuable contributions of the Myositis Discovery Programme Consumer Panel to the development of this survey.

**Consent to Participate:**

- I confirm that I have read the project information and if I have any questions I can contact the principal investigator.
- My participation is voluntary and I am free to withdraw at any time.
- Any data I provide will be treated securely and kept confidential.
- I agree to take part in this survey.

By partaking in this survey, you are providing consent to use this information for research purposes and agreeing to the above statements.

**Section 1: Details about you and your condition**

**Question 1: Has a doctor diagnosed you with myositis?**

- Yes
- No
- I’m not sure

**Question 2: What type of myositis do you have?**

- Dermatomyositis
- Juvenile dermatomyositis
- Polymyositis
- Inclusion body myositis
- Anti-synthetase syndrome
- Overlap myositis (myositis associated with scleroderma, lupus, or mixed connective tissue disorder)
- Necrotising autoimmune myopathy (also termed immune-mediated necrotising myopathy)
- Other
- I’m not sure

*For participants who selected “Other” – a free text option appears allowing them to specify more details*

**Question 3:** **In which country did you receive your diagnosis of myositis?**
*A drop down list of countries presented with participant being able to choose one country from the list.*

**Question 4: What sort of doctor(s) was/were involved in diagnosis of your condition? (Please select all that apply)**

- Rheumatologist
- Neurologist
- Internal medicine physician
- Respiratory physician
- Primary care doctor (also known as a general practitioner, GP)
- Rehabilitation physician
- I’m not sure
- Other

*For participants who selected “Other” – a free text option appears allowing them to specify more details*

**Question 5: When did you first experience symptoms of myositis? (please enter an exact date, even if it is an estimate)**

*An option is available for participants to enter in a date*

**Question 6: When did you receive your diagnosis of myositis? (please enter an exact date, even if it is an estimate)**

*An option is available for participants to enter in a date*

**Section 2: Muscle biopsy details**

**Question 7: How many muscle biopsies have you had?**

- 0
- 1
- 2
- 3
- 4
- >4

*For those who answered “0” to Question 7, the following drop-down question appeared for further clarification*

**Question 7a: Why haven’t you had a muscle biopsy? (Please select all that apply)**

- My doctor didn’t offer one or advised me that it wasn’t necessary
- I was too unwell to have a muscle biopsy
- I was worried about the risks of a muscle biopsy
- The wait for muscle biopsy was too long
- I couldn’t afford a muscle biopsy financially
- I had other commitments that meant I couldn’t have a muscle biopsy (e.g. work, caring responsibilities)
- The reason for the muscle biopsy was not adequately explained to me
- I’m not sure
- Other

*For those who selected a value greater than 1 in answer to Question 7, the following drop-down question appeared for further clarification*

**Question 7b: Why have you had more than one muscle biopsy performed? (Please select all that apply)**

- The initial biopsy didn’t provide enough information about my condition
- For research purposes
- For monitoring of my condition
- I’m not sure
- Other

**Question 8: When did you have your first muscle biopsy performed (please enter an exact date, even if it is an estimate)***An option is available for participants to enter in a date*

**Question 9: From which muscles did you have your first muscle biopsy taken? (If more than one muscle was biopsied during the same procedure then please select all that apply)**

- Thigh muscle (quadriceps)
- Back of thigh muscle (hamstrings)
- Shin muscle (tibialis anterior)
- Calf muscle (gastrocnemius)
- Upper arm muscle (biceps or triceps)
- Shoulder muscle (deltoid)
- I’m not sure
- Other

*For participants who selected “Other” – a free text option appears allowing them to specify more details*

**Question 10: Were you asleep or awake during your first muscle biopsy?**

- Asleep
- Awake
- I’m not sure/I can’t remember

*For participants who answered “Asleep” to Question 10, a further drop-down question appeared for further clarification*

**Question 10a: Did you require intubation (insertion of a breathing tube) during your first muscle biopsy?**

- Yes
- No
- I’m not sure

**Question 11:** **What sort of clinician or doctor performed your first muscle biopsy?**

- Surgeon
- Radiologist
- Rheumatologist
- Neurologist
- Other
- I’m not sure

*For participants who selected “Other” – a free text option appears allowing them to specify more details*

**Question 12: How as your sample of muscle obtained?**

- A surgical incision or cut through the skin only
- A needle through the skin (with or without a small cut to the skin)
- Conchotome forceps (scissor-like forceps) with a small cut to the skin
- Other
- I’m not sure

**Question 13:** **Was your biopsy performed as a:**

- Day procedure (i.e. you did not stay overnight in hospital)
- Inpatient admission (i.e. you spent at least a night in hospital)

**Question 14: Did the muscle biopsy reveal useful information about your condition?**

- Yes
- No
- I’m not sure

**Question 15: Did you experience any of the following negative sensations DURING your muscle biopsy procedure? (Please select all that apply)**

- Yes, pain
- Yes, pressure that was uncomfortable
- Yes, light-headedness or fainting
- Yes, anxiety
- Yes, other sensation
- No, I didn’t experience any negative sensations
- I’m not sure/I don’t remember

**Question 16: Did you experience any of the following negative outcomes AFTER your muscle biopsy? (Please select all that apply)**

- Bleeding that required medical assistance
- Bruising that required medical assistance
- Pain for which I required pain medications
- Pain that lasted more than 3 days
- Numbness at/near the site of the biopsy
- Increased weakness in the biopsied muscle lasting more than 3 weeks
- Scarring that bothers me
- I didn’t experience any negative outcomes following biopsy
- Other

*For participants who selected “Other” – a free text option appears allowing them to specify more details*

**Question 17:** **How long is the scar you have as a result of your first muscle biopsy?**

- I don’t have a visible scar
- <1cm
- Between 1-2cm
- Between 2-3cm
- >3cm

**Question 18: When did you resume your normal daily activities following muscle biopsy?**

- 1 day after biopsy
- 2 days after biopsy
- 3 days after biopsy
- >3 days after biopsy

**Question 19: Did you need to take time off paid work specifically to recover from your first muscle biopsy?**

- Yes
- No, I did not need to take time off paid work
- No, I was not in paid work at the time

*For participants who answered “Yes” to Question 19, a free-text option appeared to seek further clarification about how long they required time off pain work to recover from their first muscle biopsy.*

**Question 20: Was your muscle biopsy experience:**

- Better than expected
- As expected
- Worse than expected

**Section 3: Receiving and obtaining information about muscle biopsy**

**Question 21: Do you feel that you were given adequate information and opportunitiy to ask questions about the risks and benefits of muscle biopsy?**

- Yes
- No

**Question 22: The literature suggests that muscle biopsies are safe procedures. However, we are interested in your thoughts and beliefs. What are/were your biggest concerns about having a muscle biopsy? (Please select all that apply)**

- Pain
- Wound infection
- Scar
- Damage to muscles or nerves
- Increased weakness
- Complicaitons of a general anaesthetic
- Taking time off work
- Financial cost of muscle biopsy procedure
- Transport to and from the muscle biopsy
- Having to stay in hospital
- I don’t/didn’t have any concerns
- Other

*For participants who selected “Other” – a free text option appears allowing them to specify more details*

**Question 23: Did you consult any of the following resources for information about muscle biopsy procedures? (Please select all that apply)**

- The doctor who diagnosed me with myositis
- A doctor who performs muscle biopsies
- My primary care doctor (GP)
- A patient support group
- Social media
- An internet search (e.g. Google)
- Friends and/or family
- I did not seek out any information regarding muscle biopsies
- Other

*For participants who selected “Other” – a free text option appears allowing them to specify more details*

**Section 4: Donating muscle for research**

**Question 24: Were you invted to donate a piece of your muscle for research purposes?**

- Yes
- No

**Question 25:** **Would you agree to a future muscle biopsy if it was required for research purposes?**

- Yes
- No
- I’m not sure

**Question 25: If you are willing, please elaborate:**

*The option to provide a free-text answer was offered.*

**Question 26: Do you have any other comments or general feedback regarding your muscle biopsy experience that you would like to share with the study team?**

*The option to provide a free-text answer was offered.*

**Question 27:** **Please provide your email address below if you are happy to be contacted by the study team about further research projects on myositis (optional). Providing your email will also enable us to send you a summary of the results of this study.**
